# A deep learning model for carotid plaques detection based on CTA images: a two stepwise early-stage clinical validation study

**DOI:** 10.3389/fneur.2024.1480792

**Published:** 2025-01-13

**Authors:** Zhongping Guo, Ying Liu, Jingxu Xu, Chencui Huang, Fandong Zhang, Chongchang Miao, Yonggang Zhang, Mengshuang Li, Hangsheng Shan, Yan Gu

**Affiliations:** ^1^Department of Radiology, The First People’s Hospital of Lianyungang, Lianyungang Clinical College of Nanjing Medical University, Lianyungang, China; ^2^Department of Research Collaboration, R&D Center, Beijing Deepwise & League of PHD Technology Co., Ltd., Beijing, China; ^3^Deepwise Artificial Intelligence (AI) Lab, Deepwise, Beijing, China

**Keywords:** computed tomography angiography, artificial intelligence, head and neck, carotid plaque, deep learning

## Abstract

**Objective:**

To develop a deep learning (DL) model for carotid plaque detection based on CTA images and evaluate the clinical application feasibility and value of the model.

**Methods:**

We retrospectively collected data from patients with carotid atherosclerotic plaques who underwent continuous CTA examinations of the head and neck at a tertiary hospital from October 2020 to October 2022. The model combined ResUNet with the Pyramid Scene Parsing Network (PSPNet) to enhance plaque segmentation. Patient plaques were divided into training, validation, and testing sets in a ratio of 7:1.5:1.5. We analyzed recall (lesion-level sensitivity), sensitivity (patient-level), and precision to evaluate the model’s diagnostic performance for carotid plaques. The two stepwise early-stage clinical validation study (Comparison study and Model-human study) was used to simulate real clinical plaque diagnostic scenarios.

**Results:**

In total, 647 patients were included in the dataset, including 475 for training, 86 for validation, and 86 for testing. The DL model based on CTA images showed good precision in plaque diagnosis (validation set: precision = 80.49%, sensitivity = 90.70%, recall = 84.62%; test set: precision = 78.37%, sensitivity = 91.86%, recall = 84.58%). In addition, subgroup analysis of the plaque was carried out in the test set. The model had high accuracy in identifying plaques at different locations (Recall: 83.72, 76.32, 89.25, and 83.02%) and with different morphologies (Recall: 86.03, 79.17%). This model also analyzed the results of different types of plaques and showed good to moderate plaque diagnostic accuracy for different plaque types (Recall: 70.00, 86.87, 84.29%). Especially, in the clinical application scenario analysis, the model’s diagnostic results for plaques were found to be higher than those of 4 out of 6 radiologists (*p* < 0.001). Furthermore, in Model-human Real Clinical Scenarios study, we found that the model improved the radiologists’ sensitivity in diagnosing plaques. Additionally, the model’s diagnostic time for plaques (6 s) was found to be significantly shorter than that all of radiologists (*p* < 0.001).

**Conclusion:**

This AI model demonstrated strong clinical potential for carotid plaque detection with improved clinician diagnostic performance, shortening time, and practical implementation in real-world clinical cases.

## Introduction

Stroke remains the leading cause of death and disability worldwide (1). Clinically, atherosclerosis of the carotid artery is closely related to stroke and cerebral infarction (2). Moreover, atherosclerosis of the carotid artery is mainly manifested by the formation of atherosclerotic plaques in the carotid artery, and the presence of plaques can lead to stenosis or occlusion of the carotid artery in the corresponding segments, thereby resulting in the emergence of ischemic clinical symptoms. In addition, vulnerable plaques are prone to rupture and dislodgement due to their unstable composition, which can cause clinical symptoms and increase the burden on patients. Therefore, early assessment and clinical intervention are crucial for the prevention of cerebrovascular events in patients with vulnerable plaques.

Arteriosclerotic plaques in the carotid artery can be diagnosed by a variety of imaging methods, such as ultrasound, CTA, MRI, DSA etc., which can achieve qualitative and quantitative analysis of plaque components, detect and monitor the progress of the plaque at the early stage, provide medical staff with the opportunity to implement preventive measures, and reduce the risk of stroke and related complications.

CTA is less operator-dependent than ultrasound, and imaging is faster than MRI, and has been widely used to evaluate carotid plaques. Traditionally, the detection of carotid plaques relies on manual inspection of the images by an experienced radiologist for a detailed assessment of all vascular structures, which takes a relatively long time. In addition, CTA image analysis also requires expertise in cerebrovascular imaging, which relies on the subjective diagnosis of the reader. Therefore, the analysis process of the plaque is time-consuming and subjective, and artificial intelligence (AI) is of utmost importance.

In recent years, AI algorithms have been increasingly applied to improve the efficiency and precision of image analysis and have shown high performance as well as quick and precise evaluation on large quantities of data with reduced personal errors. Chen et al. (3) reported a study in which an AI model outperformed a visual assessment model by a reader, thus suggesting that AI algorithms can be used as a clinical tool to provide objective insights for disease diagnosis (4). Deep learning (DL) is an AI form that uses artificial neural networks to generate automatic predictions directly from image data, which can realize image detection, classification, reconstruction and other tasks (5), and has been widely and successfully applied in the field of medicine to assist in the clinical diagnosis and prognosis of diseases (6). DL methods are based on various artificial neural networks to gain knowledge of relevant and effective features from image data, which completely changes the detection and segmentation of plaques compared with machine learning. Previous studies (7, 8) have shown the potential of various DL models on the detection of coronary artery stenosis and plaque quantification, and several DL models of carotid plaque based on MRI (9–12) and ultrasound (13–15) have great potential.

Due to its widespread application and rapid imaging speed, CTA has been widely utilized to assess carotid plaques. In this study, a DL model is proposed based on carotid plaque on CTA image architecture to build an automatic plaque detection model using the CTA images. The subgroup analysis of plaque location, morphology and types was conducted on the test set, with the objective of verifying the accuracy of the model different subgroups. Furthermore, a two stepwise early-stage clinical validation study was done with the intention of providing assistance in the clinical assessment of carotid plaque.

## Materials and methods

### Participants

Patients with atherosclerotic plaques in the carotid artery who continuously underwent CTA examination of head and neck from October 2020 to October 2022 at one tertiary hospital were selected.

Inclusion criteria were: (1) patients suspected of cerebrovascular disease (typical symptoms including ischemic cerebrovascular events) in the ipsilateral eye (transient monocular blindness or retinal infarction) or in the cerebral hemisphere (transient ischemic attack (TIA) or stroke); (2) patients diagnosed with carotid plaques by CTA; (3) age > 18 y.

Exclusion criteria were: (1) patients with a history of interventional or surgical treatment, such as carotid artery stenting or carotid endarterectomy; (2) patients with carotid hemangioma or carotid vascular malformation; (3) patients with unclear or incomplete CTA images for subsequent image processing; (4) missing clinical data.

Patients were randomized into a training set, validation set, and test to the ratio of 7:1.5:1.5 (Supplementary Figure 1). Because this was a retrospective study, informed consent of the participants was waived after ethical review (KY-20220726002-01).

### CTA image acquisition

#### Equipment and reagents

A Siemens Somatom Definition Flash dual source CT scanner was selected, and the scanning parameters were set as follows: current: 125 mA, voltage: 100 kV, collimation: 16 × 0.6 mm, and layer thickness: 0.75 mm. In addition, an intravenous indwelling needle, double barrel syringe, iodixanol contrast agent (320 mg I/ml, Jiangsu Hengrui Pharmaceuticals Co., Ltd., Jiangsu, China), and 40 mL normal saline were prepared.

#### Scanning protocol

During the examination, the patient was placed in a supine position with his head tilted backward and scanned when the breath was held at the end of exhalation. The scanning range was from the aortic arch to the skull top, and the scanning direction was from the foot to the head. Post-contrast enhanced scanning was performed after routine scanning, with a scanning duration of 8.5 ± 1.5 s. Imaging of all patients was performed by the same experienced imaging technician who advised patients not to swallow before the scan and their heads were immobilized during scanning.

### Model development

#### Criteria for plaque outlining

Each image was reviewed by two radiologists who have been engaged in head and neck imaging diagnosis for more than 5 years. When two reviewers disagreed on the results, a discussion would follow to make a consensus. The plaque at the bifurcation of the carotid artery were selected, and ITK-SNAP software^1^ was employed to set the boundary of the carotid artery plaque and the region of interest from the distal end to the proximal end on the axial image (Supplementary Figure 2). Determination of plaque delineation range: the entire range involved in the plaque at the bifurcation of the common carotid artery was delineated. If the plaque was extensive, the lowest delineation was drawn to the aortic arch, and the highest delineation to the skull base. The vessels on the occluded side of the carotid artery were not delineated, but the plaques on the contralateral vessels were.

#### DL algorithms

In this study, ResUNet with the Pyramid Scene Parsing Network (PSPNet) were combined for plaque segmentation. The architecture combining ResUNet and PSPNet was designed to leverage the strengths of both models for effective plaque segmentation tasks. The 3D-CNN ResNet-50 was the main architecture of the network. The ResUNet architecture comprises an encoder, a jump connection and a decoder. The encoder employs ResNet residual blocks. In the encoder section, a series of residual blocks (ResNet-50) gradually reduces the spatial resolution and increases the depth of the feature map. We combine the encoder part of ResUNet with the Pyramid Pooling Module (PPM) module of PSPNet. The encoder part of ResUNet is combined with the Pyramid Pooling Module (PPM) of PSPNet in order to enhance the encoder by utilizing the PPM module to enhance the feature maps. In this section, a number of pooling operations are applied at different scales (1×1, 3×3, and 7×7) from the last layer of the residual module. The results of these pooling operations are then up-sampled to the dimensions of the original feature maps and spliced to the original feature map. Finally, the ResUNet decoder combines the pyramidal pooling with the final segmentation results through the convolutional layer. The encoder was used to extract high-level features from the input image, whereas a decoder incorporated a decoder inspired by the UNet structure to progressively restore resolution and fuse low-level and high-level features through skip connections, thereby preserving detailed information. PSPNet can capture global context information at different scales through pyramid pooling. This module can fuse the features of receptive fields of different sizes to improve the network’s ability to understand the overall context of the image. The decoder output of ResUNet was fused with the output of the pyramid pooling module of PSPNet, usually using element-wise addition or concatenation. This ensured effective fusion of local and global information. The output layer of the network was processed by a segmentation head, which outputs plaque prediction segmentation masks through convolutional layers and activation functions. This structure enabled the network to not only obtain high-resolution features in local areas using ResUNet, but also obtain contextual information on a global scale through PSPNet, thereby achieving better performance in plaque segmentation tasks. In practical applications, the network structure may be adjusted and optimized according to the characteristics of specific tasks and data sets.

#### Model training

First, the original CTA image was normalized to 0–1 according to the window level of 400 and the window width of 1,200. Second, for large volumetric CTA images, extract smaller patches to facilitate training and mitigate memory constraints. A patch size of 256*256*128 was used to facilitate our input data with half the overlap on the preprocessed CTA image. These preprocessing steps aimed to ensure that the CTA data were well-suited for training and evaluation with ResUNet and PSPNet architectures.

**Figure 1:**
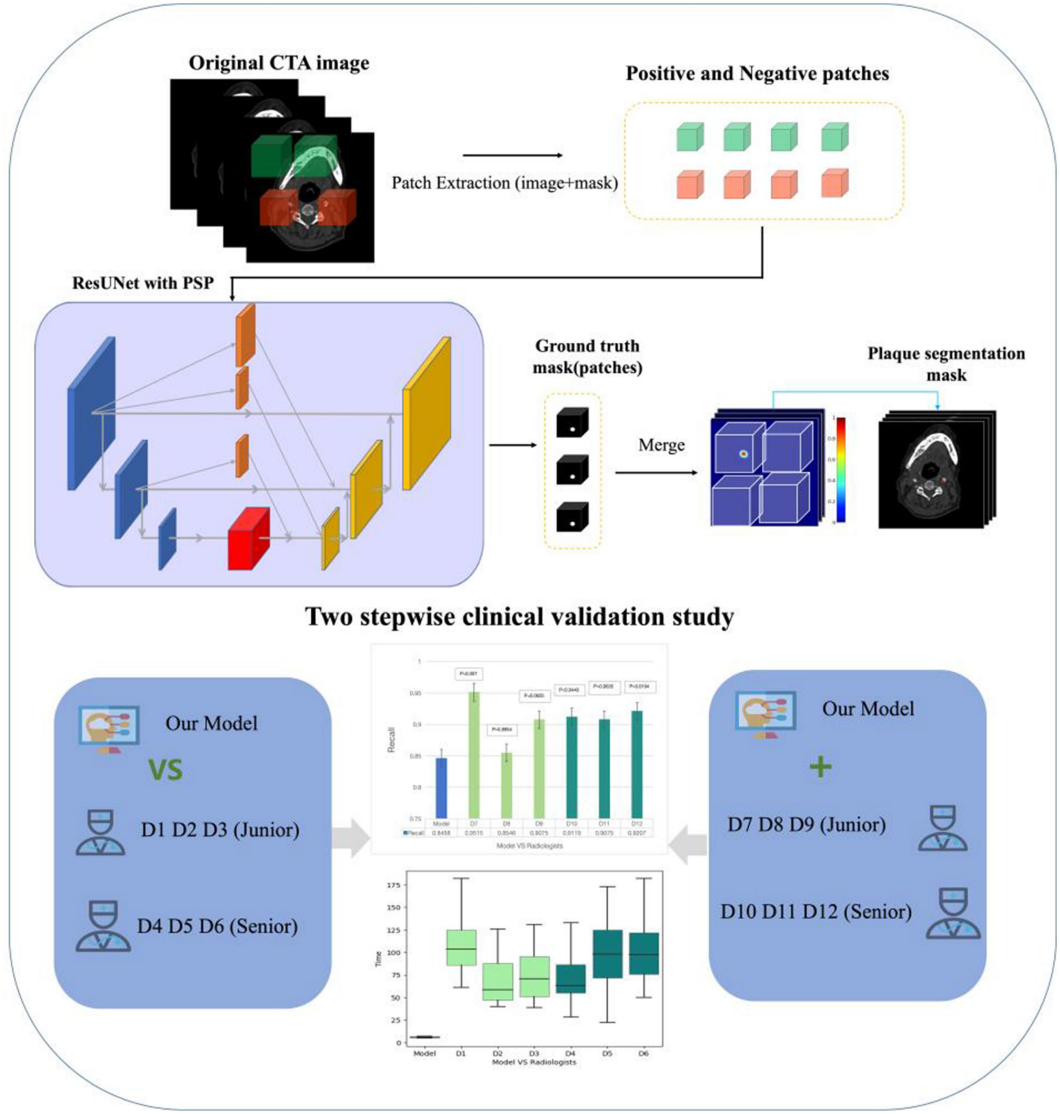
The main structure of the work. It introduces the flow of the deep learning algorithm and the two-step clinical scenario validation of the algorithm model.

The model was trained on the PyTorch framework, thereby employing a combination of ResUNet and PSP modules. The initial learning rate was set to 0.0005 with the incorporation of a warm-up strategy. The Adam optimizer was utilized with betas set to (0.9, 0.99) and a weight decay of 0.001. The loss function was a combination of Cross-Entropy (CE) loss and Dice loss, with equal weights assigned (1:1) to achieve a balanced optimization approach (Figure 1).

#### Subgroup analysis of model performance

In the test set, the location of plaques (anterior, posterior, internal, external), plaque morphology (smooth plaques, non-smooth plaques) and plaque type (soft plaque, calcified plaque, mixed plaque) on a patient’s CTA images were marked by two senior professional radiologists. If there was a disagreement, the two professionals negotiated the final decision.

##### Plaque location

The maximum cross-sectional layer of carotid plaque on the axial image was selected, and the cross-sectional lumen was divided into four 90° sectors, namely anterior wall, lateral wall, posterior wall, and medial wall. If the plaque spanned 2 sections, the area where the thickest part of the plaque was located was chosen.

##### Plaque morphology

According to the intersection surface between the plaque and the residual vessel lumen, there were two types of morphology: smooth and non-smooth. Non-smooth plaques referred to plaques with an irregular surface.

##### Plaque type

Based on the density of plaques, they are divided into three types: soft plaques, calcified plaques, and mixed plaques.

#### Model stepwise validation

##### Comparison study

We adopted the test dataset to compare the detection performance between the AI model alone and 6 radiologists. Furthermore, no clinical information or other comparative images were provided for each CTA examination. Among the 6 radiologists, 3 junior radiologists worked for less than 5 years (D1, D2, D3) and the other 3 junior radiologists worked for more than 5 years (D4, D5, D6). Radiologists diagnosed Plaque relying on their clinical expertise. The radiologists’ detection results and timing were recorded and used for comparison with the model (Figure 1).

##### Model-human real clinical scenarios study

Another 6 radiologists interpreted each CTA case with the assistance of the AI model in reading process. Furthermore, no clinical information or other comparative images were provided for each CTA examination. 3 of them were junior with working time less than 5 years, which were D7, D8, D9, and the other 3 were senior worked for more than 5 years (D10, D11, D12). Final detection results and reading time (recorded automatically) were recorded. This study was used to simulate the gain of the model for the radiologists in a real clinical scenario (Figure 1).

### Statistical analysis

Quantitative variables were utilized to express the mean ± standard deviation (SD), while categorical variables were presented as frequencies/percentages. Continuous variables were analyzed using either the Student’s t-test or the Wilcoxon test, while class-based variables were assessed using the Chi-square test or Fisher’s exact test. Model performance was evaluated on the test datasets using metrics such as recall (lesion-level sensitivity, representing the proportion of plaques correctly classified by the model), sensitivity (patient-level, representing the proportion of plaques correctly classified by the model in different patients) and precision. Furthermore, the McNemar test was used to determine if there were significant differences in sensitivity in the different data groups. A significance level of *p* < 0.05 was considered statistically significant. Statistical analysis was conducted using R software (version 3.5.2, R Foundation for Statistical Computing, Vienna, Austria).

## Results

### Clinical features

A total of 647 patients with 1982 plaques were included in the study and patients were randomly divided into training set (*N* = 475), validation set (*N* = 86), and test set (*N* = 86) according to the ratio, with the average age of 65.08 ± 9.73 years, 64.77 ± 9.41 years, and 66.94 ± 9.85 years. The patients included 221 (34.16%) females and 426 (65.84%) males, with 158 (33.26%) females and 317 (66.74%) males in the training set, 28 (32.56%) females and 58 (67.44%) males in the validation set, 35 (40.70%) females and 51 (59.30%) males in the test set (Table 1). Clinical baseline information of the patient, such as the presence of symptoms, medical history (including hypertension, diabetes), and life history (such as smoking and alcohol consumption), was also recorded in the Table 1.

**Table 1:** Clinical features.

Parameter	Training cohort	Validation cohort	Test cohort
No. of patients (*N*/%)	475 (73.42)	86 (13.29)	86 (13.29)
Mean age (y) mean ± SD	65.08 ± 9.73	64.77 ± 9.41	66.94 ± 9.85
No. of patients with symptoms (*N*/%)	356 (74.95)	55 (63.95)	58 (61.63)
Gender			
Male (*N*/%)	317 (66.74)	58 (67.44)	51 (59.30)
Female (*N*/%)	158 (33.26)	28 (32.56)	35 (40.70)
Medical history			
Hypertension (*N*/%)	327 (68.84)	58 (67.44)	27 (31.40)
Diabetes (*N*/%)	113 (23.79)	14 (16.28)	24 (27.91)
Life history			
Smoking (*N*/%)	145 (30.53)	33 (38.37)	18 (20.93)
Drinking (*N*/%)	122 (25.68)	24 (27.91)	13 (15.12)
No. of plaques (*N*)	1,521	234	227

### Model evaluation

#### Test performance of the model

**Figure 2:**
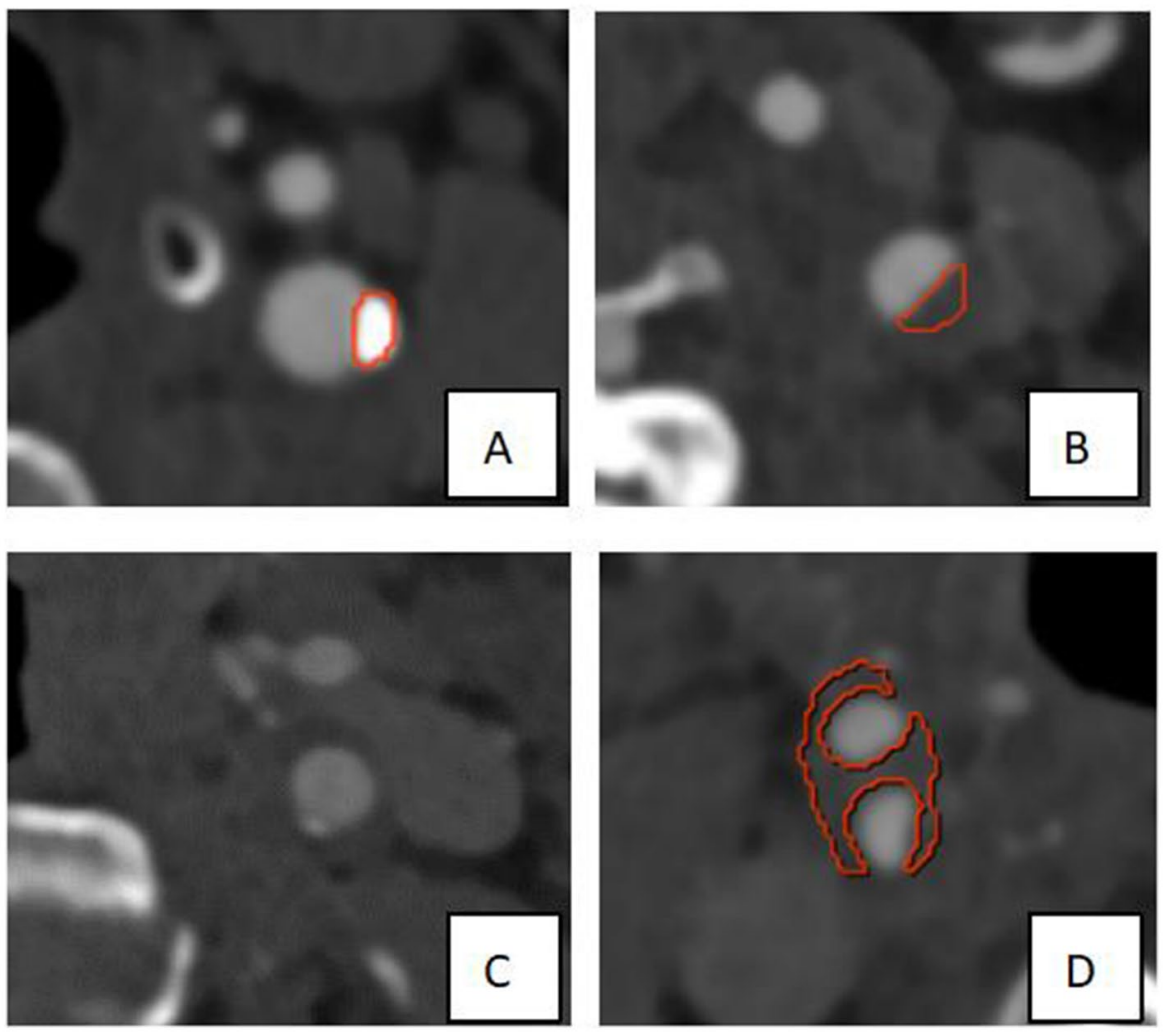
Partial presentation of modeling results. As shown in the Figure, the red border lines indicate the model’s recognition of the boundary of the plaques. **(A,B)** The models exhibited high recognition precision of calcified plaque and soft plaque on the left side of the neck. **(C)** The calcified plaque on the left side of the neck was not precisely identified, which may be due to its smaller size. **(D)** The plaque at the right carotid bifurcation was not completely and precisely identified, which may be attributed to the larger size and the location of partial plaque components near the edge.

The CTA images of 647 patients were included in the data set, including 475 for training, 86 for validation, and 86 for test. The DL model based on CTA images presented good plaque diagnosis precision (validation set: precision = 80.49%, sensitivity = 90.70%, recall = 84.62%; test set: precision = 78.37%, sensitivity = 91.86%, recall = 84.58%) (Table 2). In addition, partial presentation of modeling results were shown in Figure 2.

**Table 2:** Diagnostic results of deep learning model.

	TP	FN	FP	Count	Recall (lesion-level)	Precision	Sensitivity (patient-level)
Train	1,324	197	230	1,521	0.8705	0.8520	0.9516
Val	198	36	48	234	0.8462	0.8049	0.9070
Test	192	35	53	227	0.8458	0.7837	0.9186

#### Subgroup analysis on the results of the model in the test set

Subgroup analysis of plaques in the test set was performed to evaluate the precision of the model based on plaque location, plaque morphology and plaque type, respectively. According to the location of the plaques, four groups were made, including an anterior group, posterior group, internal group, and external group. Through comparing the plaque location identified by model with that identified manually, it was discovered that the recall of the plaque location in the anterior, posterior, internal, and external groups was 83.72, 76.32, 89.25, and 83.02%, respectively. According to the surface morphology, plaques were divided into a smooth group and non-smooth groups, and the results indicated that the recall of the plaque morphology in smooth and non-smooth groups was 86.03 and 79.17%, respectively. The model showed good lesion diagnostic accuracy for different types (soft plaque: recall = 70.00% calcified plaque: recall = 86.87%, mixed plaque: recall = 84.29%) (Table 3).

**Table 3:** Subgroup analysis on the results of the model in the test set.

	TP	FN	Count	Recall
Location				
Anterior	36	7	43	0.8372
Posterior	29	9	38	0.7632
Internal	83	10	93	0.8925
External	44	9	53	0.8302
Morphology				
Smooth	154	25	179	0.8603
Non-smooth	10	38	48	0.7917
Type				
Soft	14	6	20	0.7000
Calcified	119	18	137	0.8687
Mixed	59	11	70	0.8429

#### Model stepwise validation results

##### Comparison study

**Figure 3:**
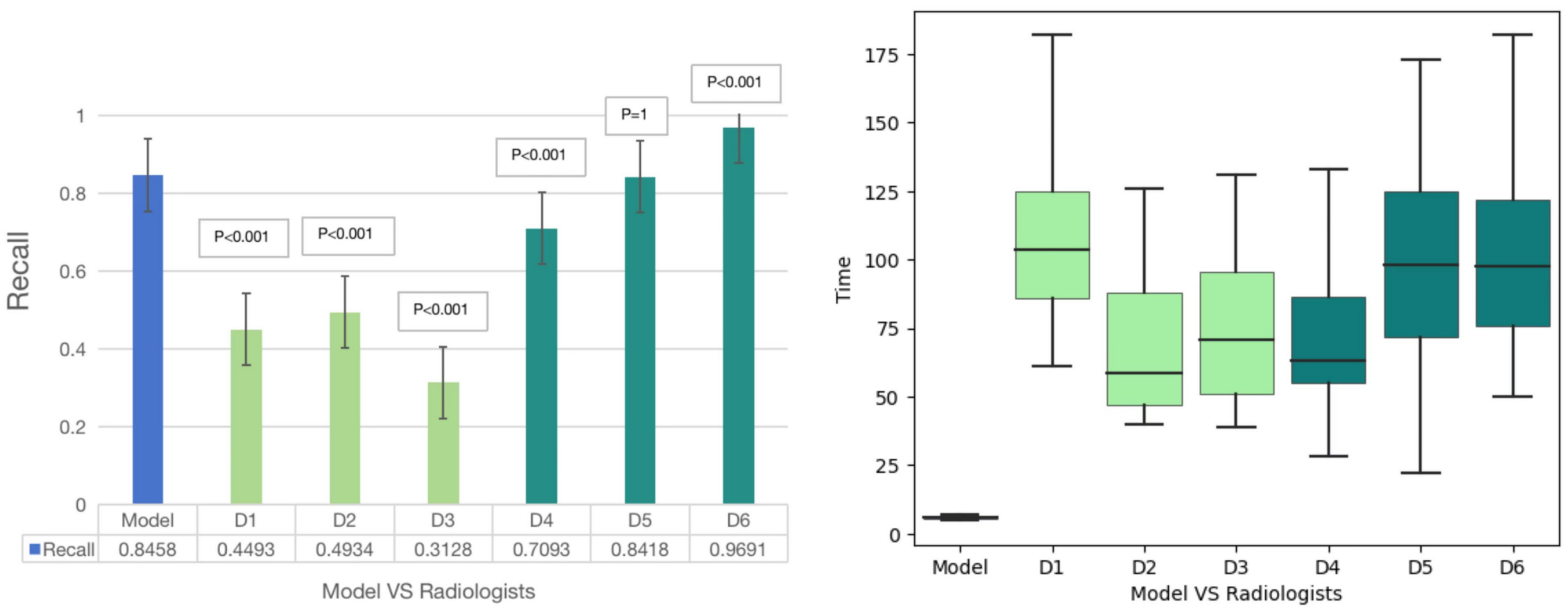
Diagnostic results of comparison study. Among 6 radiologists, only D5 radiologists having higher plaque recall values than the model, indicating that the model has higher performance in plaque diagnosis and can assist physicians in improving the accuracy of plaque diagnosis. The model diagnosis time for plaques is 6 s, significantly shorter than the diagnosis time of 6 radiologists.

For plaque diagnosis, the results showed that the recall of plaque diagnosis was low among 3 junior radiologists, and the model recall value was higher than that of 3 radiologists (*p* < 0.001); for 3 senior radiologists, the plaque diagnostic recall values were all high, with only D5 radiologists having higher plaque recall values than the model, indicating that the model has higher performance in plaque diagnosis and can assist physicians in improving the accuracy of plaque diagnosis. The model diagnosis time for plaques was 6 s, significantly shorter than the diagnosis time of 6 radiologists (97.50 ± 15.24 s) (*p* < 0.001) (Figure 3).

##### Model-human real clinical scenarios study

**Figure 4:**
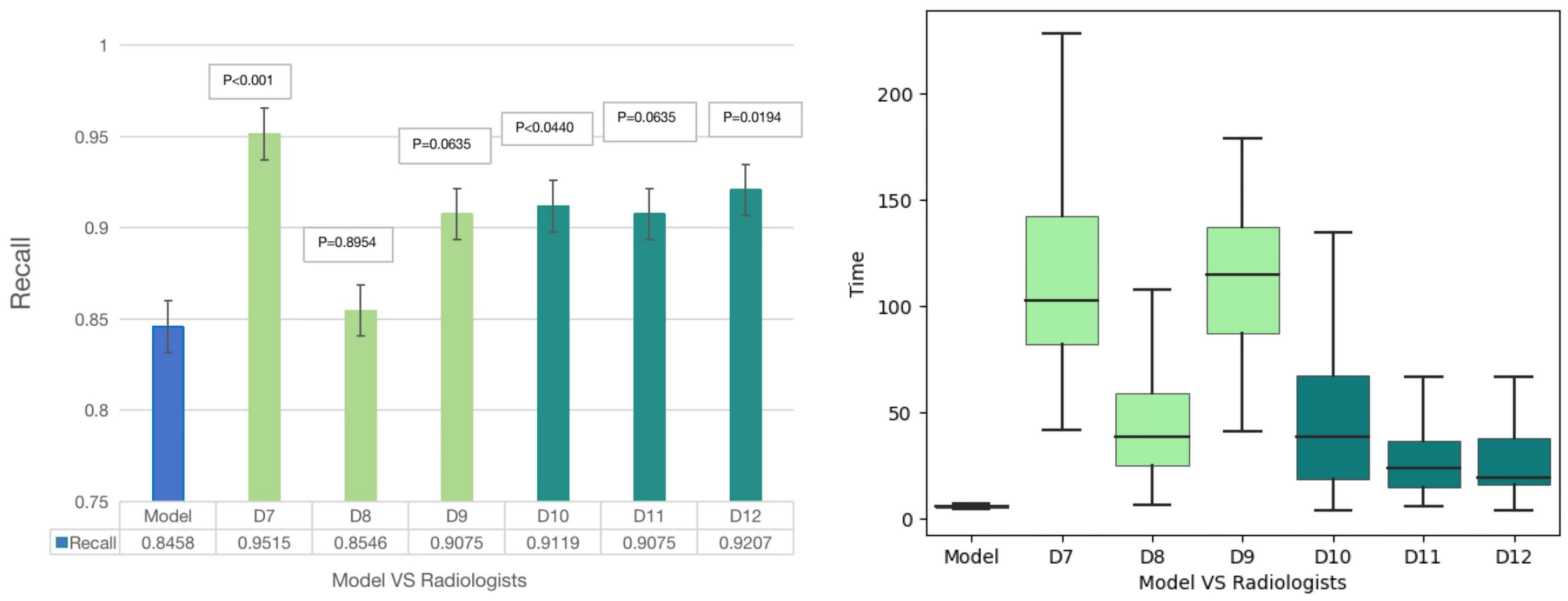
Diagnostic results of Model-human study. For plaque diagnosis, the results showed that 6 radiologists had improved diagnostic recall. The radiologists’ diagnoses (D7, D10 and D12), when aided by the model, exhibited a markedly higher degree of accuracy than model. Junior radiologist (D7) took longer to achieve a higher diagnostic performance and senior radiologists spent less time and achieved better performance. The model diagnosis time for plaques was 6 s, which was still significantly shorter than the diagnosis time of 6 radiologists.

For plaque diagnosis, the results showed that 6 radiologists (D7-D12) had improved diagnostic recall. The radiologists’ diagnoses (D7 D10 and D12), when aided by the model, exhibited a markedly higher degree of accuracy than model. Junior radiologist (D7) took longer to achieve a higher diagnostic performance. Senior radiologists spent less time and achieved better performance. The model diagnosis time for plaques was 6 s, which was still significantly shorter than the diagnosis time of 6 radiologists (74.67 ± 36.12 s) (*p* < 0.001) (Figure 4).

## Discussion

This study demonstrates that the DL model holds significant clinical potential for plaque detection. Subgroup analysis revealed high identification accuracy for plaques at different locations, different morphologies and various plaque types. Particularly in clinical scenarios, the model outperformed 4 out of 6 radiologists in plaque diagnosis. Furthermore, in real-world clinical settings, the model increased radiologists’ sensitivity and reduced the time to diagnosis (6 s, significantly faster than radiologists).

Atherosclerosis of the carotid artery is a common mechanism of ischemic stroke (16, 17). Relevant studies (18, 19) have summarized the effectiveness of DL algorithm models in assessing high-risk carotid plaque, judging plaque stability, and identifying responsible plaque. Zhu et al. (20) used DL algorithm to segment CTA images of the head and neck of 93 patients to study the plaque images. Their study included only one training set and one validation set, and did not further evaluate the algorithm’s accuracy in the test set. In this study, a new DL model was developed with a total of 647 patient images, and our findings suggest that using AI as a diagnostic aid has a positive impact on the diagnosis of carotid plaque, which is consistent with previous results (21).

Most prior studies have focused on stenosis (22), but plaque type is also a crucial factor for vulnerability. This study assessed various plaque types, revealing good to moderate diagnostic accuracy: soft plaques (recall = 70.00%), calcified plaques (recall = 86.87%), and mixed plaques (recall = 84.29%). Calcified plaques show high CT density and CT is considered as the gold standard for identifying calcification, which likely accounts for the model’s high accuracy in diagnosing them. The lower accuracy for soft plaques may be due to their density being closer to surrounding tissue, making them harder to distinguish. Additionally, the small number of soft plaques in the test set could influence performance. Plaque vulnerability is also related to morphology and distribution location (23). The model performed well in diagnosing plaques different morphologies and locations (recall: 86.03, 79.17, 83.72, 76.32, 89.25%, 83. 02%). Irregular plaques, with uneven surfaces, yielded slightly lower recall compared to smooth plaques. The study (23) have also shown that carotid plaques in the posterior wall were longer in length, with larger cross-sectional area and hardening artifacts in the surrounding skull, which may be related to the lower model recall of posterior wall plaques than plaques in other sites.

In exploring the clinical application of the model, two validation studies were conducted: a comparative study and a model-human study. These studies simulated real-world clinical scenarios, confirming the model’s value in improving plaque detection rates and reducing diagnosis time. The results align with previous research (20), suggesting that DL models can assist in plaque diagnosis and lessen radiologists’ workload. Many studies (24) have shown that AI models can outperform detecting plaques, particularly in CT-based assessments. However, most studies (25, 26) have focused on plaque segmentation, classification, and plaque-induced lumen stenosis. Few have examined DL algorithms for plaque detection in clinical settings. Our study, which emphasizes plaque detection and its clinical applicability, compared the diagnostic accuracy of the model with radiologists at various experience levels and evaluated its impact on plaque detection rates.

Current plaque analysis is time-consuming, relying heavily on expert review. DL methods automate complex tasks, such as vascular wall profiling and adaptive Hounsfield unit thresholds, which improve plaque identification. This reduces the time radiologists spend on analysis and aids clinical diagnosis. UNet is a full convolutional network semantic segmentation algorithm commonly used in imaging to automatically segment and support the diagnosis of a range of vascular diseases (24). No new is an advanced DL neural network that utilizes UNet technology with adaptive capabilities to adapt to different image properties and target structures (27–29). However, the use of UNet assisted segmentation of atherosclerotic plaques in CTA remains uncommon. A recent study (20) used the off-the-shelf algorithm nnUnet for carotid plaque segmentation. Since the network structure is not specifically designed for the patch segmentation task, the segmentation performance is not very good.

In our study, we combined ResUNet with the Pyramid Scene Parsing Network (PSPNet) for more effective patch segmentation. This hybrid architecture leverages both models’ strengths, improving segmentation accuracy. Our findings suggest that the DL algorithm holds significant potential for carotid plaque recognition, supporting its use as an auxiliary diagnostic tool. Although the model shows considerable accuracy, further validation in larger cohorts is necessary to confirm its clinical applicability. Prospective studies are warranted to assess the model’s categorical ability and evaluate its clinical impact. Ultimately, our study highlights the potential of DL-based carotid CTA models to improve diagnostic accuracy and efficiency.

This study has some limitations. First, this study was developed based on data from a single center. In a subsequent study, multicenter data with a larger sample size will be collected to update the model, aiming to improve the precision and generalizability of the model. Second, the relatively small sample size of this study may limit the generalizability of our findings. Although we have attempted to reduce this limitation by carefully selecting our cohorts and applying rigorous statistical methods, future studies with larger and more diverse cohorts are needed to validate and extend the study results. Finally, the retrospective enrollment used in this study was artificially determined, which may cause some selection bias and limit the generalizability of the results.

## Conclusion

In conclusion, in this study, ResUNet and PSPNet were combined to segment carotid plaque CTA images. The results showed that the DL model has high precision in plaque recognition as well as in recognizing plaques with different locations, morphologies and types. This AI model demonstrated strong clinical potential for carotid plaque detection with improved clinician diagnostic performance, shortening time, and practical implementation in real-world clinical cases. Future studies with larger data sets from multi-centers will be performed to further improve the application robustness of this plaque diagnosis model.

## Data Availability

The raw data supporting the conclusions of this article will be made available by the authors, without undue reservation.

## Footnotes

^1^ www.ITK-SNAP.org

## Supplementary material

Supplementary Figure 1 The flowchart of the patient inclusion process.

Supplementary Figure 2 The Figure showed the manual segmentation examples of soft plaque, mixed plaque, and calcified plaque, respectively.
