# Supplementary figures and images for: A deep learning model for carotid plaques detection based on CTA images: a two stepwise early-stage clinical validation study
Source: Front Neurol. 2025 Jan 13;15:1480792. doi: 10.3389/fneur.2024.1480792 (PMC11769795; doi:10.3389/fneur.2024.1480792)

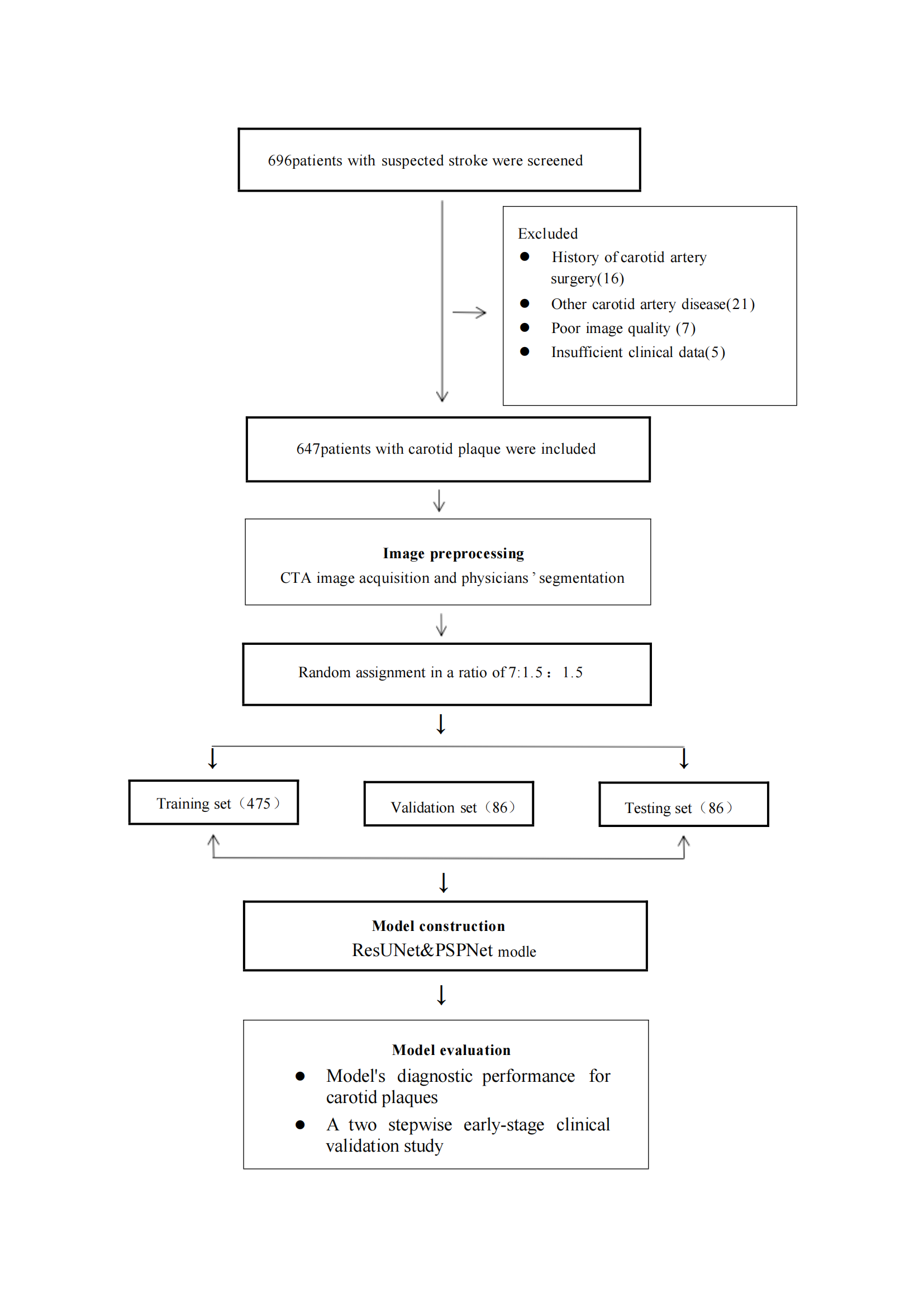

Supplement: Supplementary Figure 1 — The flowchart of the patient inclusion process. [file Image_1.PNG]

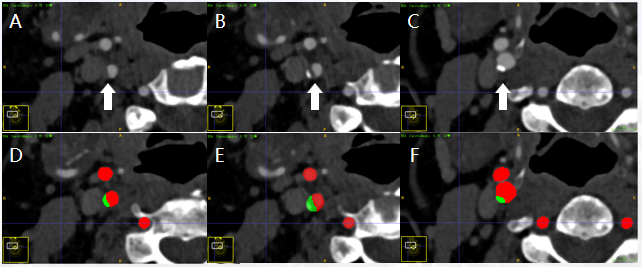

Supplement: Supplementary Figure 2 — The Figure showed the manual segmentation examples of soft plaque, mixed plaque, and calcified plaque, respectively. [file Image_2.PNG]
